# Supplementary material for: Lineage tracing using a Cas9-deaminase barcoding system targeting endogenous L1 elements
Source: Nat Commun. 2019 Mar 15;10:1234. doi: 10.1038/s41467-019-09203-z (PMC6420643; doi:10.1038/s41467-019-09203-z)
Supplement: Supplementary file 1 — Supplementary Information [file 41467_2019_9203_MOESM1_ESM.pdf]

Lineage tracing using a Cas9-deaminase barcoding system targeting endogenous L1 elements

Byungjin Hwang et al.

Supplementary Figures

Supplementary Figure 1. Schematic overview of the targeted deaminase system. a) After amplification using a single primer pair, edited C>T substitution patterns were encoded to serve as cell barcodes for lineage reconstruction. b) Distinct regions were targeted using two sgRNAs designed for use in this study.

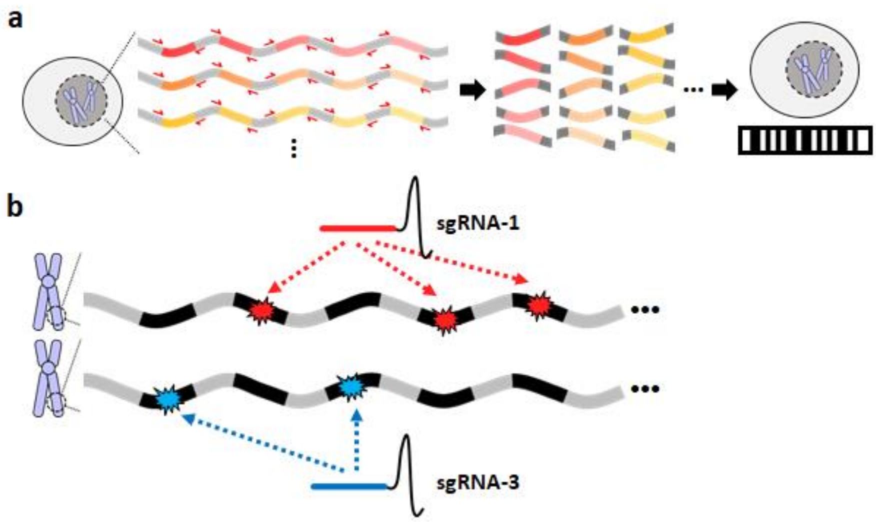

Supplementary Figure 2. Characterization of the amplified target regions. A density plot of the amplicon size (measured in fragment length inferred by alignment of the NGS reads) with two distinct peaks (left) and a representative histogram of the L1 retrotransposon subfamily distribution by decreasing order of counts (right).

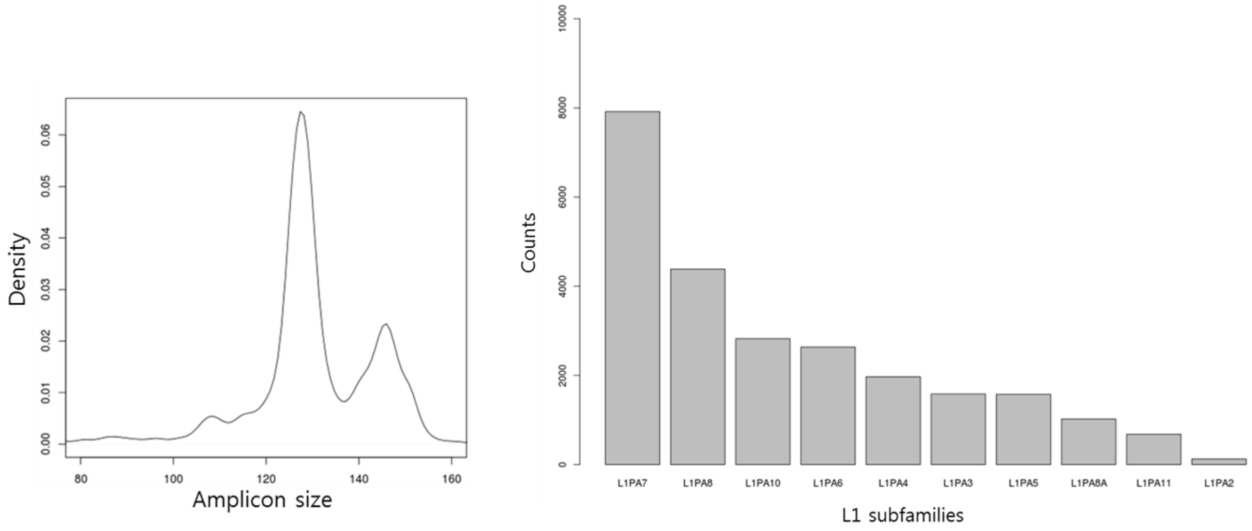

Supplementary Figure 3. Top 10 candidate sgRNAs in the targeted regions. The top 10 sgRNA candidates are shown in descending order of candidates with perfect targetable sites. The actual count is shown above the bar graph. The two candidates used in this study were sgRNA-1 and sgRNA-3. We excluded sgRNA-2 because of the sequence similarity to sgRNA-1 (nearly identical sites would be targeted as there was only a one base difference of the spacer region) between the spacer sequence of the two sgRNAs.

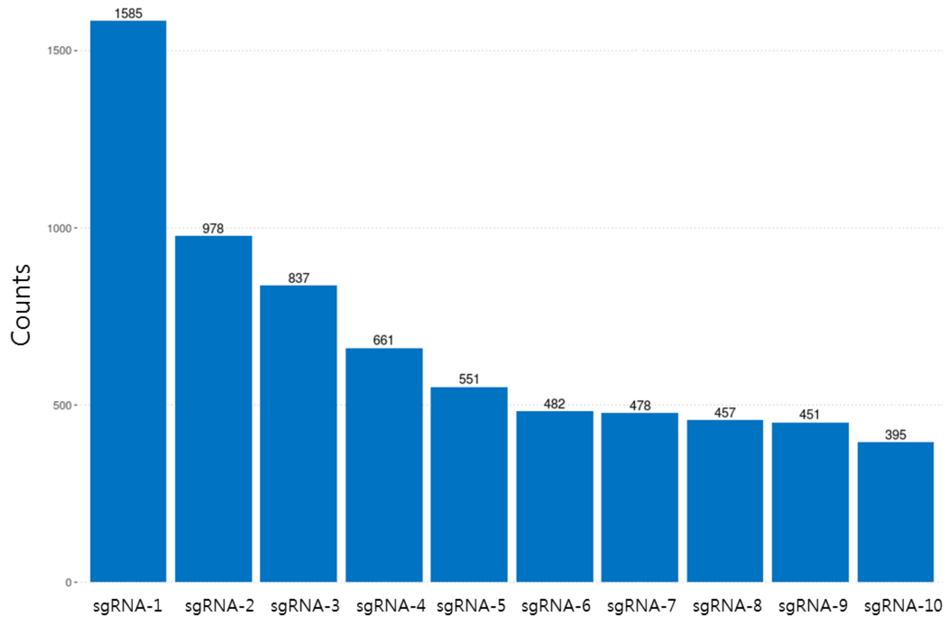

Supplementary Figure 4. Design of Targeted deaminase vector.

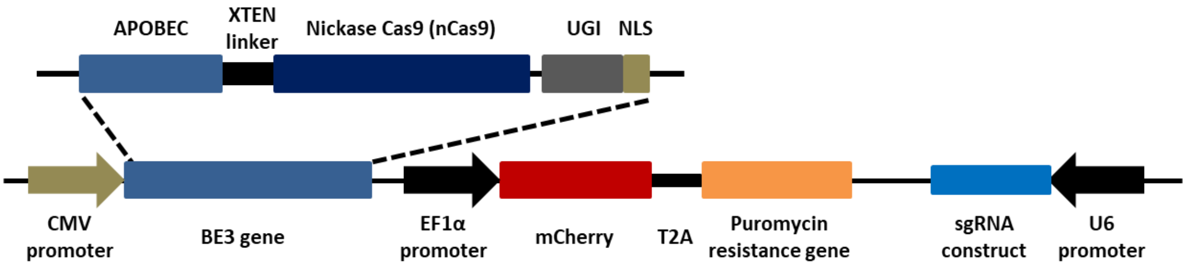

Supplementary Figure 5. Comparison of editing efficiency in the spacer region. All the 'C' positions (second to sixth) in non-target Cs in the spacer region showed little or no detectable editing compared to the known target region (4-8 positions from the PAM distal end). The first 'C' is located at the 8th position of the spacer region for the sgRNA-1 design (**Supplementary Figure 2**). Mutated C's are underlined and Non-mutated C's are indicated inside the plotting box. sgRNA-1 sequence is shown below with the C's indicated in the bold with corresponding order in bracket from the left.

[sgRNA-1 : 5'-ATGGGTG **C(1)** AG **C(2)** AAA **C(3)** **C(4)** A **C(5)** **C(6)** A-3']

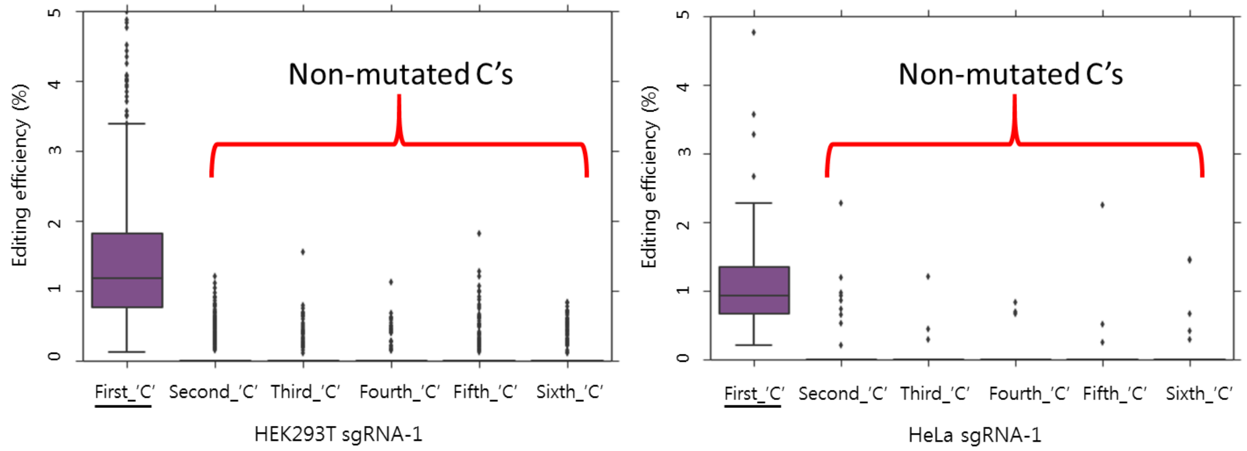

Supplementary Figure 6. Schematic overview of *in vitro* tree expansion in the bulk experiment. First, the barcoding system containing BE3 and sgRNA is transduced into cells using lentivirus and selected using puromycin. Single cells are isolated from mCherry-expressing cells in individual wells using FACS. Isolated positive cells are expanded for 2–3 weeks, leveraging a mosaic effect rather than making a homogeneous population. This facilitates reconstruction of the lineage with minimal loss of accuracy. Some of the expanded cells are used for gDNA extraction and barcode amplification. Each cell barcode is then detected at the population level using NGS. The rest of the expanded cells are subjected for isolation of single cells using FACS, and these are expanded as in the previous procedure. The process is repeated over several generations, and cell barcodes are continually detected during this process. Reconstruction of the cell lineage is performed using the obtained cell barcodes.

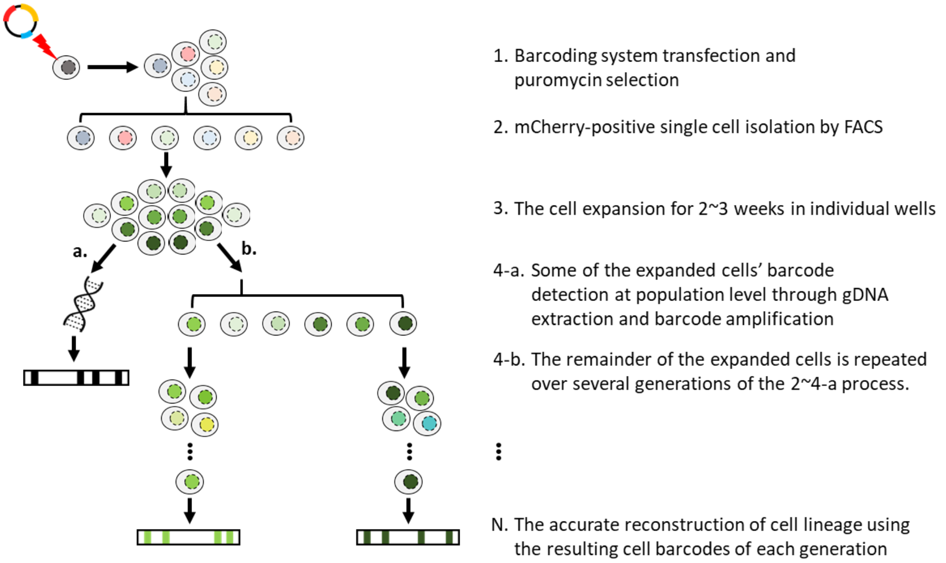

Supplementary Figure 7. In vitro tree expansion experiment for the HeLa cell line. The four major clades for sgRNA-3 are colored differently (3-1, 3-3, 3-6, 3-8). Subsequent child nodes from the parent nodes are colored with respect to the major clade. The misplaced nodes are represented by a red arrow. The red solid line connects the incorrectly placed mother-daughter node, and the dotted line indicates the correct mother-daughter node connection.

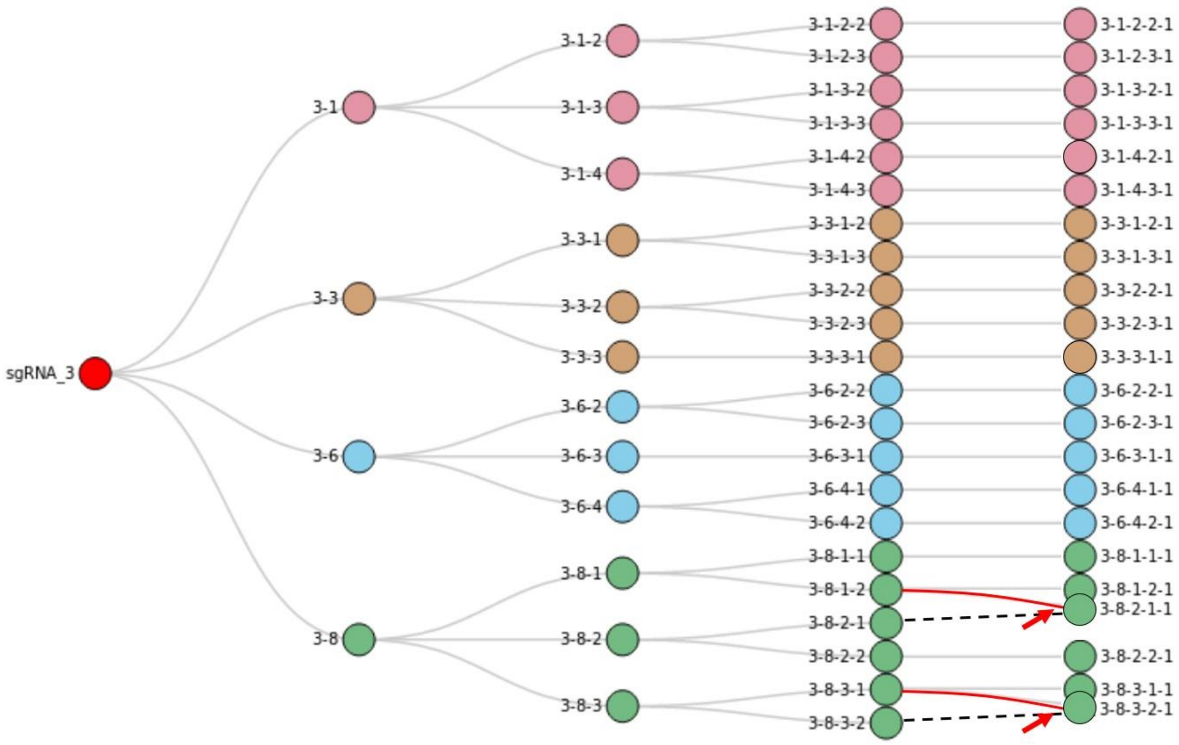

Supplementary Figure 8. Barcode representation of in vitro bulk cell experiments (from top, HEK293T and HeLa cells). Rows correspond to nodes for each clade. The clade to which each row (node) belongs is shown to the left; node numbers shown in **Figure 2** and **Supplementary Figure 7** are shown to the right for each row. The "depth" of the tree is indicated by a color gradient (lighter shades correspond to deeper tree nodes in **Figure 2b** and **Supplementary Figure 7**, and node numbers shown in red font indicate misplaced nodes). Columns correspond to editing sites in the sgRNA-3 design. Boxes shown in black dotted lines indicate cell barcodes from misplaced nodes.

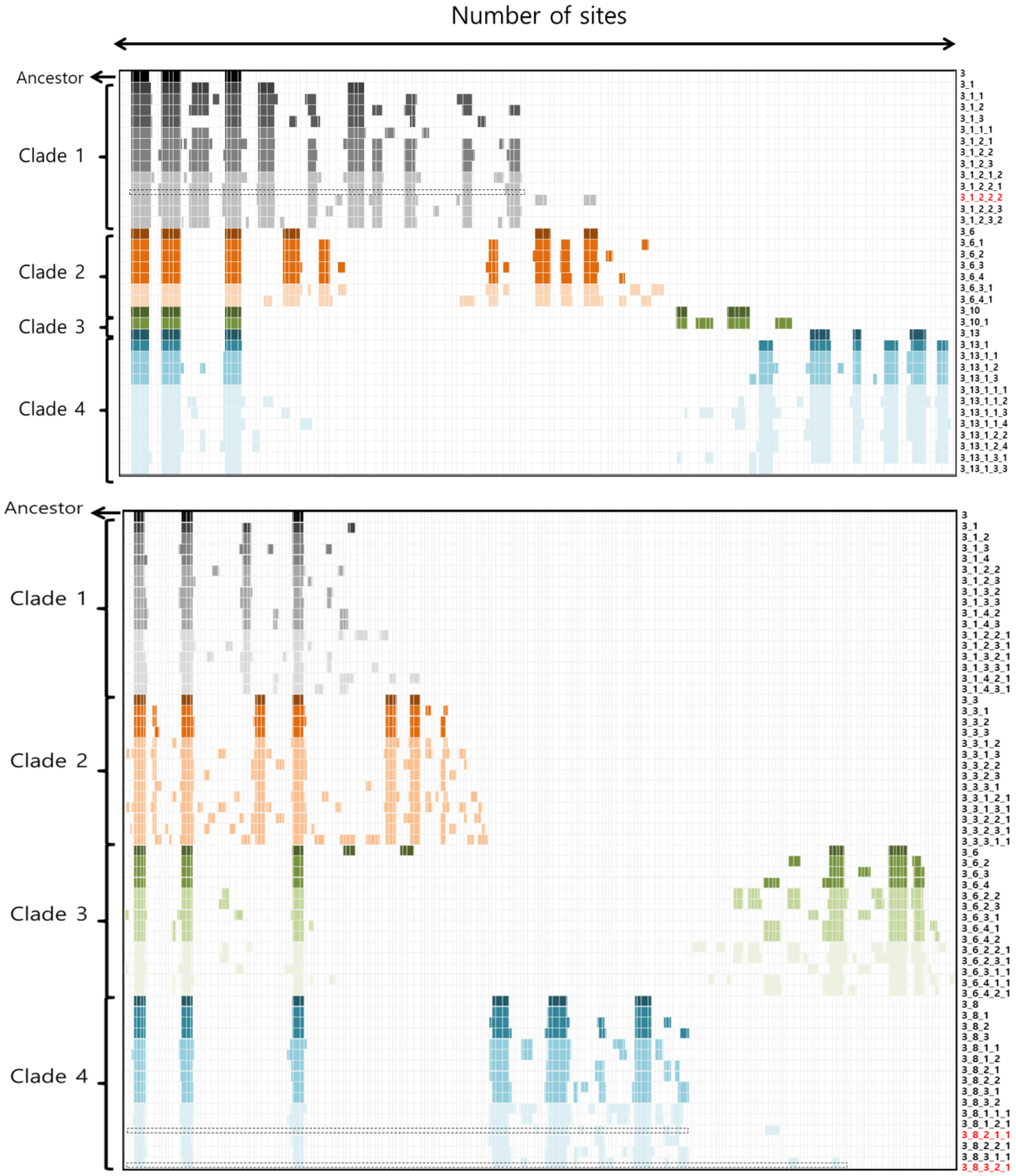

Supplementary Figure 9. Comparison of the sequencing coverage statistics between the PCR and WGA results. Lorenz plot of the single-cell sequencing library (sgRNA-3) preparation. A single-cell was picked and either PCR amplified or subjected to WGA before library preparation for sequencing. The grey dotted line (*y*=*x*) represents perfect uniformity of coverage.

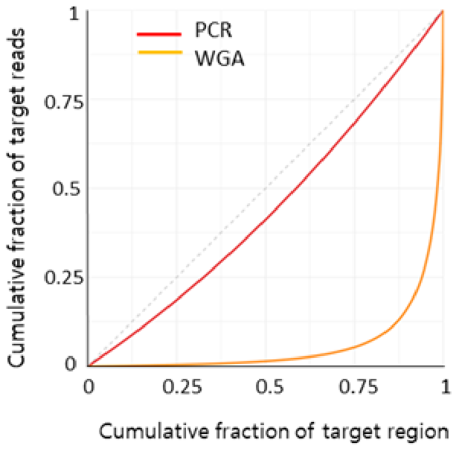

Supplementary Figure 10. An experimental expansion of single-cell video tracking. Two additional single-cell trees (sgRNA-3) are shown below. Two major clades are shown for each tree (different branch colors for different major clades). For the right tree, Cell_1_1_1_1 could not be placed correctly due to marker dropout (zero cell barcode).

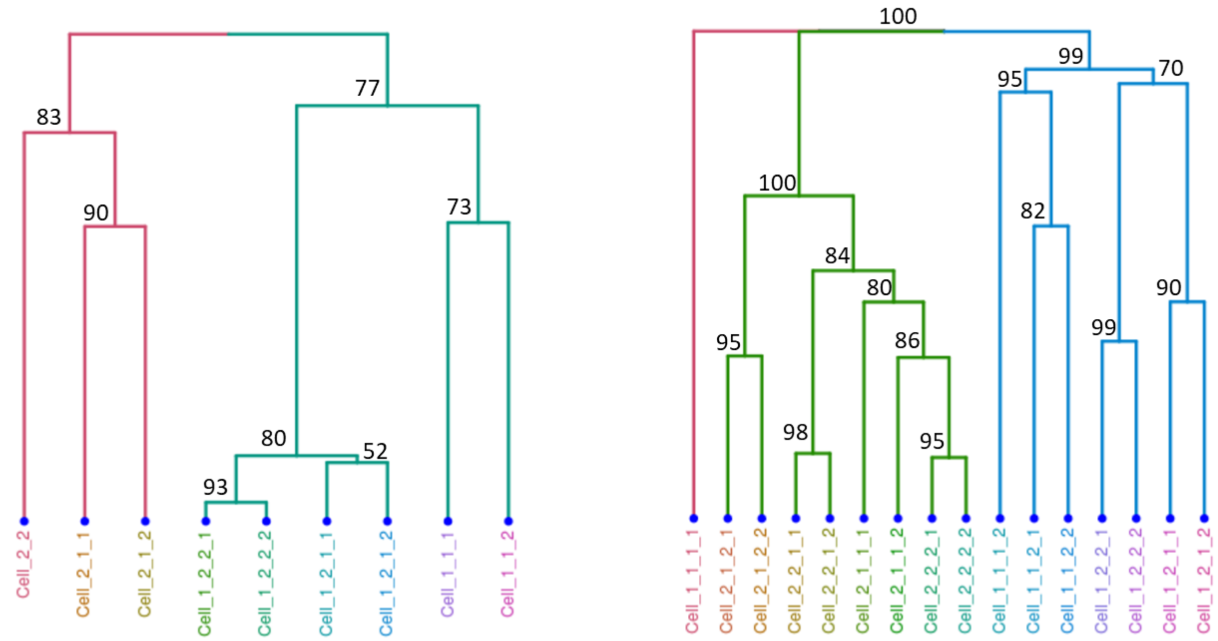

Supplementary Figure 11. Editing dynamics of sgRNA-3 measured at different time points in HEK293T cells. To estimate the editing rate, the curve was fitted (exponential function) using the average value of the two replicates.

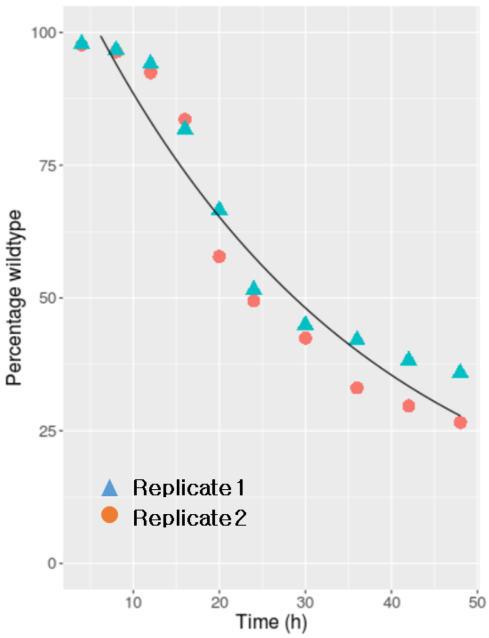

Supplementary Figure 12. Simulated tree reconstruction using sgRNA-3 with different parameters. Performance was evaluated by *in silico* experiments using the Cophenetic Correlation with replicates (error bars indicate standard deviation, n=10)

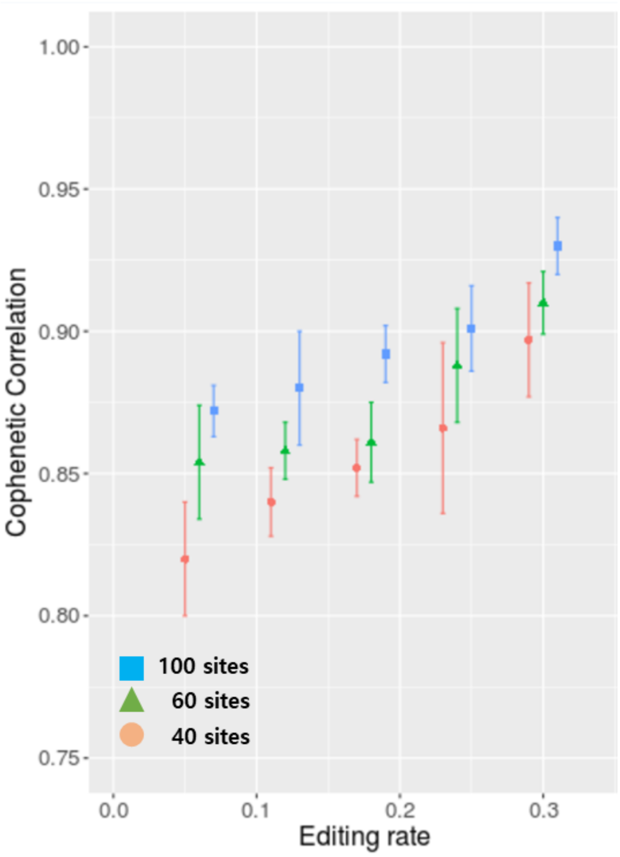

Supplementary Figure 13. Simulation of site editing bias (N=800). The site editing bias is simulated with 800 targets for comparison with an sgRNA-3 design (837 sites). We applied an empirically determined upper bound of the editing bias = 10% (~10% of the sites tend to saturate faster).

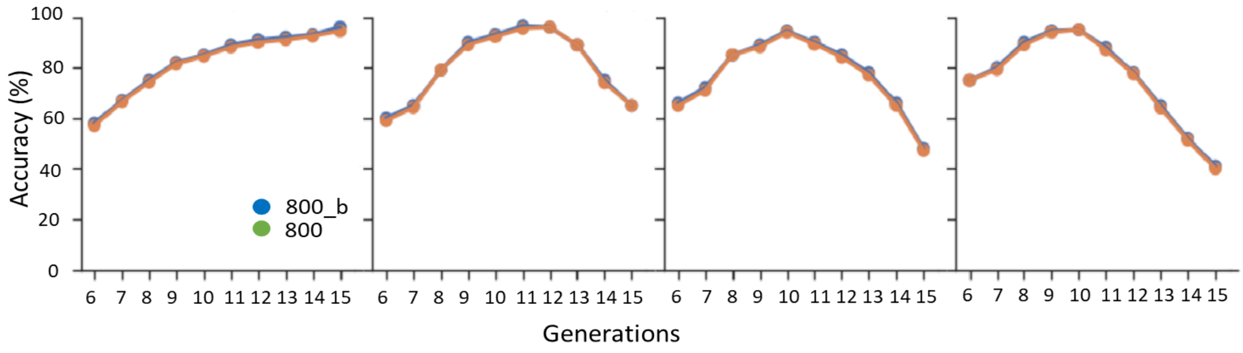

Supplementary Tables

Supplementary Table 1. Oligonucleotide used for plasmid vector construction.

Primers	Sequences (5' to 3')
L1 site for	ACACTCTTTCCCTACACGACGCTCTTCCGATCTNNNNNNNNNNNNNNNNACACAGGGAGGGGAACAT
L1 site rev	GACTGGAGTTCAGACGTGTGCTCTTCCGATCTTGCCATGGTGGTTTGCT
sgRNA-1 sgRNA for	TTTCTTGGCTTTATATATCTTGTGGAAAGGACGAAACACCGATGGGTGCAGCAAACCACCA
sgRNA-1 sgRNA rev	GACTAGCCTTATTTTAACTTGCTATTTCTAGCTCTAAAACTGGTGGTTTGCTGCACCCATC
sgRNA-3 sgRNA for	TTTCTTGGCTTTATATATCTTGTGGAAAGGACGAAACACCGGAAATACCTAATGTAGATGA
sgRNA-3 sgRNA rev	GACTAGCCTTATTTTAACTTGCTATTTCTAGCTCTAAAACTCATCTACATTAGGTATTTCC

Supplementary Table 2. Summary of coverage statistics. Summary statistics of sgRNA designs used in the study for the bulk cell line experiment. Vehicle control refers to empty gRNA expression vectors. The average breadth of coverage (>20×) for base 'C' in the target region was ~96% for HEK293T and ~100% for the HeLa cell experiments.

Sample ID	Ontarget (%)	Breadth of coverage (%)			
		1X	5X	10X	20X
HEK293T_vehicle	100	87.9937	85.5214	84.1119	81.5152
HEK293T_sgRNA-1	99.8645	87.9187	85.4802	84.0915	81.304
HEK293T_sgRNA-3	99.8771	88.1069	85.5644	84.266	81.7976
HeLa_vehicle	99.8283	85.7387	81.8852	78.7776	77.7832
HeLa_sgRNA-1	99.7969	86.4339	83.1277	80.7696	76.41
HeLa_sgRNA-3	99.8139	85.1579	80.8516	77.4631	76.5252

Supplementary Note

Supplementary Note. Sequence of Targeted deaminase vector.

ACTTACGGTAAATGGCCCGCCTGGCTGACCGCCCAACGACCCCCGCCCATTGACGTCAATAATGACGT
ATGTTCCCATAGTAACGCCAATAGGGACTTTCCATTGACGTCAATGGGTGGAGTATTTACGGTAAACT
GCCCACTTGGCAGTACATCAAGTGTATCATATGCCAAGTACGCCCCCTATTGACGTCAATGACGGTAA
ATGGCCCGCCTGGCATTATGCCCAGTACATGACCTTATGGGACTTTCCTACTTGGCAGTACATCTACG
TATTAGTCATCGCTATTACCATGGTGATGCGGTTTTGGCAGTACATCAATGGGCGTGGATAGCGGTTT
GACTCACGGGGATTTCCAAGTCTCCACCCCATTGACGTCAATGGGAGTTTGTTTTGGCACCAAAATCA
ACGGGACTTTCCAAAATGTCGTAACAACTCCGCCCCATTGACGCAAATGGGCGGTAGGCGTGTACGGT
GGGAGGTCTATATAAGCAGAGCTGGTTTAGTGAACCGTCAGATCCGCTAGAGATCCGCGGCCGCTAAT
ACGACTCACTATAGGGAGAGCCGCCACCATGAGCTCAGAGACTGGCCCAGTGGCTGTGGACCCCACAT
TGAGACGGCGGATCGAGCCCCATGAGTTTGAGGTATTCTTCGATCCGAGAGAGCTCCGCAAGGAGACC
TGCCTGCTTTACGAAATTAATTGGGGGGGCCGGCACTCCATTTGGCGACATACATCACAGAACACTAA
CAAGCACGTCGAAGTCAACTTCATCGAGAAGTTCACGACAGAAAGATATTTCTGTCCGAACACAAGGT
GCAGCATTACCTGGTTTCTCAGCTGGAGCCCATGCGGCGAATGTAGTAGGGCCATCACTGAATTCCTG
TCAAGGTATCCCCACGTCACTCTGTTTATTTACATCGCAAGGCTGTACCACCACGCTGACCCCCGCAA
TCGACAAGGCCTGCGGGATTTGATCTCTTCAGGTGTGACTATCCAAATTATGACTGAGCAGGAGTCAG
GATACTGCTGGAGAAACTTTGTGAATTATAGCCCGAGTAATGAAGCCCACTGGCCTAGGTATCCCCAT
CTGTGGGTACGACTGTACGTTCTTGAACTGTACTGCATCATACTGGGCCTGCCTCCTTGTCTCAACAT
TCTGAGAAGGAAGCAGCCACAGCTGACATTCTTTACCATCGCTCTTCAGTCTTGTCATTACCAGCGAC
TGCCCCCACACATTCTCTGGGCCACCGGGTTGAAAAGCGGCAGCGAGACTCCCGGGACCTCAGAGTCC
GCCACACCCGAAAGTGATAAAAAGTATTCTATTGGTTTAGCCATCGGCACTAATTCCGTTGGATGGGC
TGTCATAACCGATGAATACAAAGTACCTTCAAAGAAATTTAAGGTGTTGGGGAACACAGACCGTCATT
CGATTAAAAAGAATCTTATCGGTGCCCTCCTATTCGATAGTGGCGAAACGGCAGAGGCGACTCGCCTG
AAACGAACCGCTCGGAGAAGGTATACACGTCGCAAGAACCGAATATGTTACTTACAAGAAATTTTTAG
CAATGAGATGGCCAAAGTTGACGATTCTTTCTTTCACCGTTTGGAAGAGTCCTTCCTTGTCGAAGAGG
ACAAGAAACATGAACGGCACCCCATCTTTGGAAACATAGTAGATGAGGTGGCATATCATGAAAAGTAC
CCAACGATTTATCACCTCAGAAAAAAGCTAGTTGACTCAACTGATAAAGCGGACCTGAGGTTAATCTA
CTTGGCTCTTGCCCATATGATAAAGTTCCGTGGGCACTTTCTCATTGAGGGTGATCTAAATCCGGACA
ACTCGGATGTCGACAAACTGTTCATCCAGTTAGTACAAACCTATAATCAGTTGTTTGAAGAGAACCCT
ATAAATGCAAGTGGCGTGGATGCGAAGGCTATTCTTAGCGCCCGCCTCTCTAAATCCCGACGGCTAGA
AAACCTGATCGCACAATTACCCGGAGAGAAGAAAAATGGGTTGTTCGGTAACCTTATAGCGCTCTCAC
TAGGCCTGACACCAAATTTTAAGTCGAACTTCGACTTAGCTGAAGATGCCAAATTGCAGCTTAGTAAG
GACACGTACGATGACGATCTCGACAATCTACTGGCACAAATTGGAGATCAGTATGCGGACTTATTTTT
GGCTGCCAAAAACCTTAGCGATGCAATCCTCCTATCTGACATACTGAGAGTTAATACTGAGATTACCA
AGGCGCCGTTATCCGCTTCAATGATCAAAAGGTACGATGAACATCACCAAGACTTGACACTTCTCAAG
GCCCTAGTCCGTCAGCAACTGCCTGAGAAATATAAGGAAATATTCTTTGATCAGTCGAAAAACGGGTA
CGCAGGTTATATTGACGGCGGAGCGAGTCAAGAGGAATTCTACAAGTTTATCAAACCCATATTAGAGA
AGATGGATGGGACGGAAGAGTTGCTTGTAAAACTCAATCGCGAAGATCTACTGCGAAAGCAGCGGACT
TTCGACAACGGTAGCATTCCACATCAAATCCACTTAGGCGAATTGCATGCTATACTTAGAAGGCAGGA
GGATTTTTATCCGTTCCTCAAAGACAATCGTGAAAAGATTGAGAAAATCCTAACCTTTCGCATACCTT
ACTATGTGGGACCCCTGGCCCGAGGGAACTCTCGGTTCGCATGGATGACAAGAAAGTCCGAAGAAACG
ATTACTCCATGGAATTTTGAGGAAGTTGTCGATAAAGGTGCGTCAGCTCAATCGTTCATCGAGAGGAT
GACCAACTTTGACAAGAATTTACCGAACGAAAAAGTATTGCCTAAGCACAGTTTACTTTACGAGTATT

TCACAGTGTACAATGAACTCACGAAAGTTAAGTATGTCACTGAGGGCATGCGTAAACCCGCCTTTCTA
AGCGGAGAACAGAAGAAAGCAATAGTAGATCTGTTATTCAAGACCAACCGCAAAGTGACAGTTAAGCA
ATTGAAAGAGGACTACTTTAAGAAAATTGAATGCTTCGATTCTGTCGAGATCTCCGGGGTAGAAGATC
GATTTAATGCGTCACTTGGTACGTATCATGACCTCCTAAAGATAATTAAAGATAAGGACTTCCTGGAT
AACGAAGAGAATGAAGATATCTTAGAAGATATAGTGTTGACTCTTACCCTCTTTGAAGATCGGGAAAT
GATTGAGGAAAGACTAAAAACATACGCTCACCTGTTCGACGATAAGGTTATGAAACAGTTAAAGAGGC
GTCGCTATACGGCTGGGGACGATTGTCGCGGAAACTTATCAACGGGATAAGAGACAAGCAAAGTGGT
AAAACTATTCTCGATTTTCTAAAGAGCGACGGCTTCGCCAATAGGAACTTTATGCAGCTGATCCATGA
TGACTCTTTAACCTTCAAAGAGGATATACAAAAGGCACAGGTTTCCGGACAAGGGGACTCATTGCACG
AACATATTGCGAATCTTGCTGGTTCGCCAGCCATCAAAAAGGGCATACTCCAGACAGTCAAAGTAGTG
GATGAGCTAGTTAAGGTCATGGGACGTCACAAACCGGAAAACATTGTAATCGAGATGGCACGCGAAAA
TCAAACGACTCAGAAGGGGCAAAAAAACAGTCGAGAGCGGATGAAGAGAATAGAAGAGGGTATTAAAG
AACTGGGCAGCCAGATCTTAAAGGAGCATCCTGTGGAAAATACCCAATTGCAGAACGAGAAACTTTAC
CTCTATTACCTACAAAATGGAAGGGACATGTATGTTGATCAGGAACTGGACATAAACCGTTTATCTGA
TTACGACGTCGATCACATTGTACCCCAATCCTTTTTGAAGGACGATTCAATCGACAATAAAGTGCTTA
CACGCTCGGATAAGAACCGAGGGAAAAGTGACAATGTTCCAAGCGAGGAAGTCGTAAAGAAAATGAAG
AACTATTGGCGGCAGCTCCTAAATGCGAAACTGATAACGCAAAGAAAGTTCGATAACTTAACTAAAGC
TGAGAGGGGTGGCTTGTCTGAACTTGACAAGGCCGGATTTATTAAACGTCAGCTCGTGGAAACCCGCC
AAATCACAAAGCATGTTGCACAGATACTAGATTCCCGAATGAATACGAAATACGACGAGAACGATAAG
CTGATTCGGGAAGTCAAAGTAATCACTTTAAAGTCAAAATTGGTGTCGGACTTCAGAAAGGATTTTCA
ATTCTATAAAGTTAGGGAGATAAATAACTACCACCATGCGCACGACGCTTATCTTAATGCCGTCGTAG
GGACCGCACTCATTAAGAAATACCCGAAGCTAGAAAGTGAGTTTGTGTATGGTGATTACAAAGTTTAT
GACGTCCGTAAGATGATCGCGAAAAGCGAACAGGAGATAGGCAAGGCTACAGCCAAATACTTCTTTTA
TTCTAACATTATGAATTTCTTTAAGACGGAAATCACTCTGGCAAACGGAGAGATACGCAAACGACCTT
TAATTGAAACCAATGGGGAGACAGGTGAAATCGTATGGGATAAGGGCCGGGACTTCGCGACGGTGAGA
AAAGTTTTGTCCATGCCCCAAGTCAACATAGTAAAGAAAACTGAGGTGCAGACCGGAGGGTTTTCAAA
GGAATCGATTCTTCCAAAAAGGAATAGTGATAAGCTCATCGCTCGTAAAAAGGACTGGGACCCGAAAA
AGTACGGTGGCTTCGATAGCCCTACAGTTGCCTATTCTGTCCTAGTAGTGGCAAAAGTTGAGAAGGGA
AAATCCAAGAAACTGAAGTCAGTCAAAGAATTATTGGGGATAACGATTATGGAGCGCTCGTCTTTTGA
AAAGAACCCCATCGACTTCCTTGAGGCGAAAGGTTACAAGGAAGTAAAAAAGGATCTCATAATTAAAC
TACCAAAGTATAGTCTGTTTGAGTTAGAAAATGGCCGAAAACGGATGTTGGCTAGCGCCGGAGAGCTT
CAAAAGGGGAACGAACTCGCACTACCGTCTAAATACGTGAATTTCCTGTATTTAGCGTCCCATTACGA
GAAGTTGAAAGGTTCACCTGAAGATAACGAACAGAAGCAACTTTTTGTTGAGCAGCACAAACATTATC
TCGACGAAATCATAGAGCAAATTTCGGAATTCAGTAAGAGAGTCATCCTAGCTGATGCCAATCTGGAC
AAAGTATTAAGCGCATACAACAAGCACAGGGATAAACCCATACGTGAGCAGGCGGAAAATATTATCCA
TTTGTTTACTCTTACCAACCTCGGCGCTCCAGCCGCATTCAAGTATTTTGACACAACGATAGATCGCA
AACGATACACTTCTACCAAGGAGGTGCTAGACGCGACACTGATTCACCAATCCATCACGGATTATAT
GAAACTCGGATAGATTTGTCACAGCTTGGGGGTGACTCTGGTGGTTCTACTAATCTGTCAGATATTAT
TGAAAAGGAGACCGGTAAGCAACTGGTTATCCAGGAATCCATCCTCATGCTCCCAGAGGAGGTGGAAG
AAGTCATTGGGAACAAGCCGGAAAGCGATATACTCGTGCACACCGCCTACGACGAGAGCACCGACGAG
AATGTCATGCTTCTGACTAGCGACGCCCCTGAATACAAGCCTTGGGCTCTGGTCATACAGGATAGCAA
CGGTGAGAACAAGATTAAGATGCTCTCTGGTGGTTCTCCCAAGAAGAAGAGGAAAGTCTAACAGCAGA
GATCCAGTTTATCGATGAGTAATTCATACAAAAGGACTCGCCCCTGCCTTGGGGAATCCCAGGGACCG
TCGTTAAACTCCCACTAACGTAGAACCCAGAGATCGCTGCGTTCCCGCCCCCTCACCCGCCCGCTCTC
GTCATCACTGAGGTGGAGAAGAGCATGCGTGAGGCTCCGGTGCCCGTCAGTGGGCAGAGCGCACATCG
CCCACAGTCCCCGAGAAGTTGGGGGGAGGGGTCGGCAATTGAACCGGTGCCTAGAGAAGGTGGCGCGG
GGTAAACTGGGAAAGTGATGTCGTGTACTGGCTCCGCCTTTTTCCCGAGGGTGGGGGAGAACCGTATA

TAAGTGCAGTAGTCGCCGTGAACGTTCTTTTTCGCAACGGGTTTGCCGCCAGAACACAGGTAAGTGCC
GTGTGTGGTTCCCGCGGGCCTGGCCTCTTTACGGGTTATGGCCCTTGCGTGCCTTGAATTACTTCCAC
GCCCCTGGCTGCAGTACGTGATTCTTGATCCCGAGCTTCGGGTTGGAAGTGGGTGGGAGAGTTCGAGG
CCTTGCGCTTAAGGAGCCCCTTCGCCTCGTGCTTGAGTTGAGGCCTGGCTTGGGCGCTGGGGCCGCCG
CGTGCGAATCTGGTGGCACCTTCGCGCCTGTCTCGCTGCTTTCGATAAGTCTCTAGCCATTTAAAATT
TTTGATGACCTGCTGCGACGCTTTTTTTCTGGCAAGATAGTCTTGTAAATGCGGGCCAAGATCTGCAC
ACTGGTATTTCGGTTTTTGGGGCCGCGGGCGGCGACGGGGCCCGTGCGTCCCAGCGCACATGTTCGGC
GAGGCGGGGCCTGCGAGCGCGGCCACCGAGAATCGGACGGGGGTAGTCTCAAGCTGGCCGGCCTGCTC
TGGTGCCTGGCCTCGCGCCGCCGTGTATCGCCCCGCCCTGGGCGGCAAGGCTGGCCCGGTCGGCACCA
GTTGCGTGAGCGGAAAGATGGCCGCTTCCCGGCCCTGCTGCAGGGAGCTCAAAATGGAGGACGCGGCG
CTCGGGAGAGCGGGCGGGTGAGTCACCCACACAAAGGAAAAGGGCCTTTCCGTCCTCAGCCGTCGCTT
CATGTGACTCCACGGAGTACCGGGCGCCGTCCAGGCACCTCGATTAGTTCTCGAGCTTTTGGAGTACG
TCGTCTTTAGGTTGGGGGGAGGGGTTTTATGCGATGGAGTTTCCCCACACTGAGTGGGTGGAGACTGA
AGTTAGGCCAGCTTGGCACTTGATGTAATTCTCCTTGGAATTTGCCCTTTTTGAGTTTGGATCTTGGT
TCATTCTCAAGCCTCAGACAGTGGTTCAAAGTTTTTTTCTTCCATTTCAGGTGTCGTGAGGATCTATT
TCCGGTGAATTCCTCGAGACTAGTTCTAGATGGTGAGCAAGGGCGAGGAGGATAACATGGCCATCATC
AAGGAGTTCATGCGCTTCAAGGTGCACATGGAGGGCTCCGTGAACGGCCACGAGTTCGAGATCGAGGG
CGAGGGCGAGGGCCGCCCCTACGAGGGCACCCAGACCGCCAAGCTGAAGGTGACCAAGGGTGGCCCCC
TGCCCTTCGCCTGGGACATCCTGTCCCCTCAGTTCATGTACGGCTCCAAGGCCTACGTGAAGCACCCC
GCCGACATCCCCGACTACTTGAAGCTGTCCTTCCCCGAGGGCTTCAAGTGGGAGCGCGTGATGAACTT
CGAGGACGGCGGCGTGGTGACCGTGACCCAGGACTCCTCCCTGCAGGACGGCGAGTTCATCTACAAGG
TGAAGCTGCGCGGCACCAACTTCCCCTCCGACGGCCCCGTAATGCAGAAGAAGACCATGGGCTGGGAG
GCCTCCTCCGAGCGGATGTACCCCGAGGACGGCGCCCTGAAGGGCGAGATCAAGCAGAGGCTGAAGCT
GAAGGACGGCGGCCACTACGACGCTGAGGTCAAGACCACCTACAAGGCCAAGAAGCCCGTGCAGCTGC
CCGGCGCCTACAACGTCAACATCAAGTTGGACATCACCTCCCACAACGAGGACTACACCATCGTGGAA
CAGTACGAACGCGCCGAGGGCCGCCACTCCACCGGCGGCATGGACGAGCTGTACAAGGAGGGCCGGGG
CAGCCTGCTGACCTGCGGCGACGTGGAGGAGAACCCCGGCCCCATGACCGAGTACAAGCCCACGGTGC
GCCTCGCCACCCGCGACGACGTCCCCAGGGCCGTACGCACCCTCGCCGCCGCGTTCGCCGACTACCCC
GCCACGCGCCACACCGTCGATCCGGACCGCCACATCGAGCGGGTCACCGAGCTGCAAGAACTCTTCCT
CACGCGCGTCGGGCTCGACATCGGCAAGGTGTGGGTCGCGGACGACGGCGCCGCGGTGGCGGTCTGGA
CCACGCCGGAGAGCGTCGAAGCGGGGGCGGTGTTCGCCGAGATCGGCCCGCGCATGGCCGAGTTGAGC
GGTTCCCGGCTGGCCGCGCAGCAACAGATGGAAGGCCTCCTGGCGCCGCACCGGCCCAAGGAGCCCGC
GTGGTTCCTGGCCACCGTCGGCGTCTCGCCCGACCACCAGGGCAAGGGTCTGGGCAGCGCCGTCGTGC
TCCCCGGAGTGGAGGCGGCCGAGCGCGCCGGGGTGCCCGCCTTCCTGGAGACCTCCGCGCCCCGCAAC
CTCCCCTTCTACGAGCGGCTCGGCTTCACCGTCACCGCCGACGTCGAGGTGCCCGAAGGACCGCGCAC
CTGGTGCATGACCCGCAAGCCCGGTGCCTGAACGCGTTAAGTCACCCAGCTTTCTTGTACAAAGTGGT
GATAACTCTAGAGAATTCACTCCTCAGGTGCAGGCTGCCTATCAGAAGGTGGTGGCTGGTGTGGCCAA
TGCCCTGGCTCACAAATACCACTGAGATCTTTTTCCCTCTGCCAAAAATTATGGGGACATCATGAAGC
CCCTTGAGCATCTGACTTCTGGCTAATAAAGGAAATTTATTTTCATTGCAATAGTGTGTTGGAATTTT
TTGTGTCTCTCACTCGGAAGGACATATGGGAGGGCAAATCATTTAAAACATCAGAATGAGTATTTGGT
TTAGAGTTTGGCAACATATGCCATATGCTGGCTGCCATGAACAAAGGTGGCTATAAAGAGGTCATCAG
TATATGAAACAGCCCCCTGCTGTCCATTCCTTATTCCATAGAAAAGCCTTGACTTGAGGTTAGATTTT
TTTTATATTTTGTTTTGTGTTATTTTTTTCTTTAACATCCCTAAAATTTTCCTTACATGTTTTACTAG
CCAGATTTTTCCTCCTCTCCTGACTACTCCCAGTCATAGCTGTCCCTCTTCTCTTATGAAGATCCCTC
GACCTGCAGCCCAAAAAAAAGCACCGACTCGGTGCCACTTTTTCAAGTTGATAACGGACTAGCCTTAT
TTTAACTTGCTATTTCTAGCTCTAAAACNNNNNNNNNNNNNNNNNNNNCGGTGTTTCGTCCTTTCCAC
AAGATATATAAAGCCAAGAAATCGAAATACTTTCAAGTTACGGTAAGCATATGATAGTCCATTTTAAA

ACATAATTTTAAAACTGCAAACTACCCAAGAAATTATTACTTTCTACGTCACGTATTTTGTACTAATA
TCTTTGTGTTTACAGTCAAATTAATTCTAATTATCTCTCTAACAGCCTTGTATCGTATATGCAAATAT
GAAGGAATCATGGGAAATAGGCCCTCTTCCTGCCCGACCTT

CMV promoter
BE3 gene
EF1α promoter
mCherry gene
Puromycin resistance gene
U6 promoter + sgRNA construct
